# The Experience of and Needs for Exergames in Older Adults With Mild Cognitive Impairment: Qualitative Interview Study

**DOI:** 10.2196/53631

**Published:** 2025-06-17

**Authors:** Xi Chen, Dian Jiang, Hongting Ning, Lina Wu, Yifei Chen, Chi Zhang, Ruotong Peng, Yishu Zhu, Hui Feng

**Affiliations:** 1 Xiangya School of Nursing, Central South University Changsha China; 2 School of Nursing, Hunan University of Chinese Medicine Changsha China; 3 Sun Yat-sen University School of Nursing Guangzhou China; 4 Shanxi Technology and Business University Taiyuan China

**Keywords:** exergaming, older adults, mild cognitive impairment, experience, qualitative study

## Abstract

**Background:**

As a novel intervention method that combines exercise and games, exergames have demonstrated a positive impact on enhancing the cognitive and physical functions of older adults with mild cognitive impairment (MCI). However, there remains a dearth of knowledge and evidence regarding the experiences and needs of the older adult population in China with MCI about exergames.

**Objective:**

This qualitative study aimed to investigate the experience of and needs for exergames among older adults with MCI.

**Methods:**

We adopted a phenomenological methodology for this study, and conducted it at a community and nursing home in Changsha, Hunan Province, from June to August 2023. We used the purpose sampling method to conduct semistructured interviews with 21 older people with MCI. Older people with MCI were allowed to experience exergames using our preselected exergame device, the Nintendo Switch, and they were interviewed to understand their experience and needs for exergames. The interviews were recorded and transcribed verbatim, and the data were uploaded to NVivo 12 software for encoding. The corresponding text was then reviewed for data analysis. Data analysis was guided by the methodology proposed by Giorgi and was carried out simultaneously with data collection. This study’s trustworthiness was evaluated according to credibility, dependability, confirmability, and transferability criteria.

**Results:**

Overall, 21 participants (mean age 70.2, SD 7.6 y; n=17, 81% women; mean Montreal Cognitive Assessment score 18.8, SD 3.6) were interviewed. Moreover, 21 interviews were conducted. By the 18th interview, the data were saturated, and to make sure no new topics came up, we conducted 3 more interviews. The experience of older people with MCI with exergames includes five parts: their attitudes toward exergames vary, they are both entertaining and interesting, they promote physical activity and exercise, they pass the time and relieve loneliness, and their conditions of use are not restricted. The needs of older people with MCI for exergames include the desire to design older people–friendly exergames, ensure scientific validity and safety in the process of sports, provide a good gaming experience, exercise physical and cognitive function, and provide support and training.

**Conclusions:**

This study provides an interpretative understanding of the experiences and needs associated with exergames in older people with MCI, which could inform exergame development appropriate for this population and guide the implementation of exergame interventions in this population. Most older people with MCI expressed a positive attitude toward exergames, but not all were interested in them. Older people with MCI viewed exergames as both entertaining and fun, promoting physical activity and exercise, passing the time, relieving loneliness, and the conditions of use were not restricted. Exergames for older people with MCI should be older people–friendly, scientific, safe, provide a good play experience, exercise physical and cognitive function, and provide training and support. In the future, exergames should be tailored to meet the unique needs of older people with MCI, which is critical to improving their well-being.

## Introduction

### Background

Dementia is recognized as a priority global public health issue by the World Health Organization [1]. A new case of dementia is diagnosed every 3 seconds worldwide [2]. It is estimated that the global prevalence of dementia is doubling roughly every 20 years and will reach 131.5 million by 2050 [2]. Dementia costs up to US $81 billion a year in health care costs and lost income and is estimated to increase to US $2 trillion by 2030 [2]. China currently accounts for approximately one-quarter of all patients with dementia globally, making it the country with the largest number of patients with dementia [3], which needs more attention.

There is currently no successful cure for dementia. Therefore, many researchers are focusing on mild cognitive impairment (MCI), the intermediate stage between the cognitive changes caused by normal aging and the onset of dementia. This may be the best opportunity to prevent or delay the onset of dementia [4]. The prevalence of MCI is estimated to exceed one-quarter in people aged >60 years, with an increased risk associated with increasing age [5]. The conversion rate to dementia within 3 years among patients with MCI is approximately 14 times higher than that among individuals without MCI over the same period [6]. Therefore, timely and effective intervention measures should be taken for older adults with MCI.

Exergames are a combination of exercise and games, also known as active video games, which incorporate physical activity into a video game environment through body-controlled movements [7]. Exergames provide both physical and cognitive training, and the combination of these interventions has demonstrated synergistic effects [8]. Previous systematic reviews indicated that exergames are one of the most effective exercise interventions for improving health outcomes, including cognition and physical function, in older adults [9,10]. Furthermore, promising findings from studies involving patients with MCI suggest that exergames have the potential to enhance both physical and cognitive function in this population [11,12].

However, the practical implementation of most exergames for older adults is not ideal [13]. Most exergames, especially commercially available exergames, such as the Nintendo Wii or Xbox Kinect, were not specifically developed for older adults and do not consider their characteristics and preferences [14-16]. Therefore, a user-centered design is needed to develop appropriate exergames for older adults with MCI. In addition, our team recently published a systematic review and qualitative synthesis [17] of all the qualitative studies on the experience of older adults using exergames worldwide. However, it is important to note that none of these studies focused specifically on older adults in China. Given that older adults are a susceptible group that may engage with or use exergames, it is crucial to investigate their experience and needs for exergames. Therefore, to develop user-friendly, adaptive, and personalized exergames suitable for older adults with MCI in China, we conducted this qualitative study to explore their experience and needs with regard to exergames.

### Objectives

This study aimed to investigate the experience and needs of older adults with MCI in China about exergames. The findings from this study hold substantial importance in informing the design and development of exergames, as well as guiding the implementation of exergame interventions for older adults, particularly those with MCI.

## Methods

### Qualitative Approach

To achieve the purpose of the study, we used the semistructured interview method. This method of in-depth interview is an effective approach for explanatory questioning, because it allows for a deep exploration and examination of an individual’s views [18]. At the same time, to better understand the experiences and actual needs of older adults with MCI, we used the phenomenological methodology [19]. It focuses on the individual and helps the participants clarify the basic elements of personal experience [20,21]. At the same time, our approach was guided by the approach taken in the study by Giorgi [20,22], which is a systematic approach to data collection, analysis, and synthesis suitable for scientific analysis, and emphasizes the importance of researcher engagement and reflection in the aforementioned process. We used the COREQ (Consolidated Criteria for Reporting Qualitative Research) checklist [23] to ensure that this study met the recommended standards for reporting qualitative data.

### Study Setting and Patient Recruitment

From June 2023 to July 2023, we used the purpose sampling method to recruit participants in a community and nursing home in Changsha City, Hunan Province, by pasting posters, distributing leaflets, or recommending potential participants by the nursing home staff. First, members of our research team contacted interested older adults with MCI by phone to provide detailed information about the study procedures. Those who expressed interest in participating were then asked for their consent and subsequently screened for eligibility. The inclusion criteria were as follows: (1) aged ≥60 years and (2) pass the diagnostic criteria for MCI by Petersen [24] (self-reported or insider reported memory decline; scores based on the Montreal Cognitive Assessment [25] were ≤13 points for the illiterate group, ≤19 points for the primary school group, and ≤24 points for the junior high school group and higher; the score of the Activity of Daily Living Scale was ≤23 points; no clinical diagnosis of dementia). The exclusion criteria were as follows: (1) patients with serious heart, lung, kidney, and other organ diseases; (2) patients with severe limb motor dysfunction, visual impairment, and hearing impairment; and (3) patients with severe mental illness. Participants were continually recruited until data were saturated and no new topics emerged from the final interviews. Participants who were willing to participate in the semistructured interview and met the inclusion criteria were fully informed of the study objectives and procedures, and all participants provided written informed consent.

### Data Collection

All interviews were conducted by 2 trained researchers (XC and DJ) in the form of face-to-face interviews with the participants during July to August 2023. Each interview lasted 40 to 60 minutes, and the 2 researchers transcribed the recordings verbatim into text files within 24 hours of the interview. In addition, the researchers also made field notes for subsequent analysis. The researchers continued to conduct interviews until the data were saturated. Demographic questionnaires were also conducted after each interview. The interview was divided into 2 parts: the first part involved allowing older adults with MCI to experience exergames using our preselected exergame device Nintendo Switch. The second part was to interview older adults with MCI about their experience of and needs for exergames.

**Figure 1:**
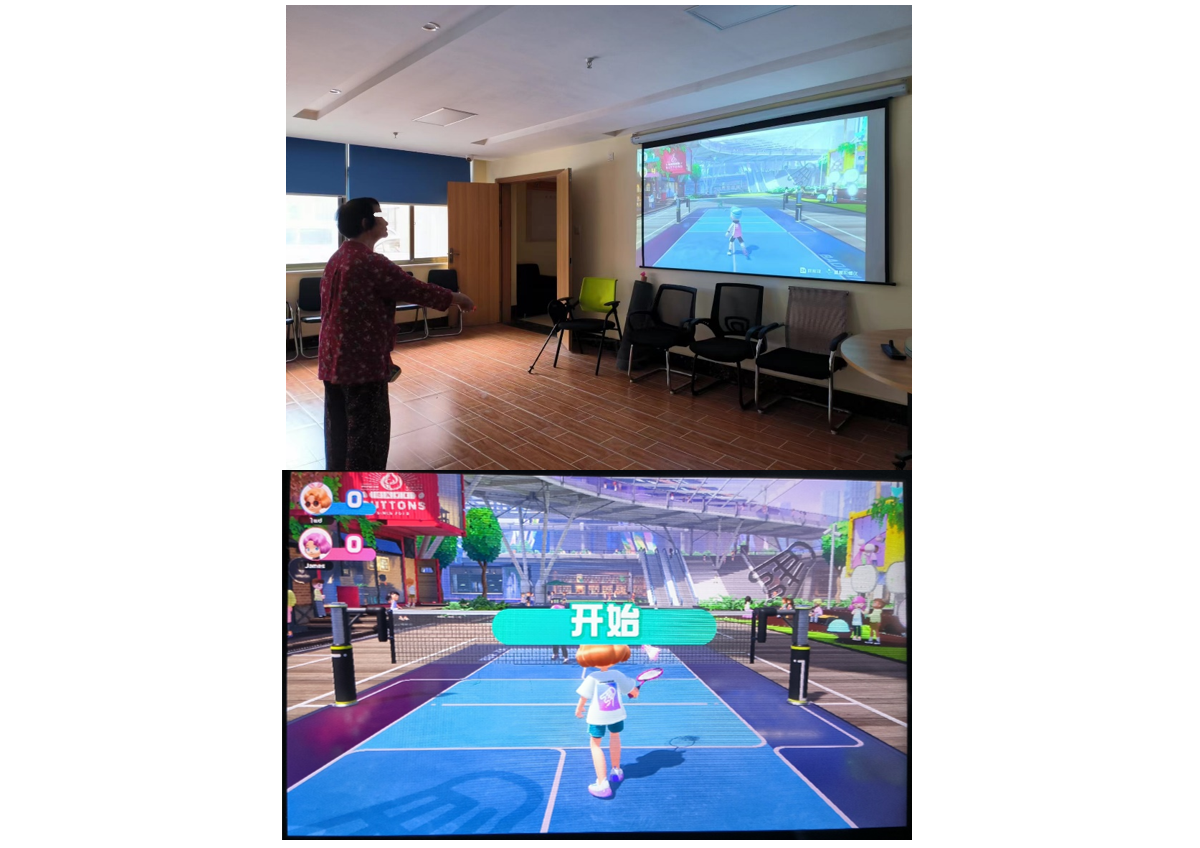
Nintendo Switch Sports.

The first part was an exergaming experience for older adults with MCI using our preselected, existing commercial exergame device Nintendo Switch. Given that older adults are a susceptible group in contact with exergames and most of them are not familiar with exergames, this study allowed older adults with MCI to play exergames for 10 to 20 minutes under the guidance of the rigorously trained research team members before the interview. The research team members promptly answered any questions from the older adults with MCI during this period to ensure familiarity and mastery of exergames. The Nintendo Switch was chosen because it has been shown to be safe, feasible, and acceptable for use in older patients [26], while also improving their cognitive and physical function and overall quality of life [27]. We invited older patients with MCI to experience a badminton game called *Nintendo Switch Sports* on the device Nintendo Switch (Figure 1). In this game, they manipulated the Joy-Con handles to execute a range of actions associated with playing badminton. These actions directly controlled the hand movements of animated characters displayed on the screen, thereby facilitating interactive engagement between older adults with MCI and the animated characters. In the process of older adults with MCI experiencing the exergame, we adjusted the settings for each participant’s motor function. In general, the games were implemented in a standing position. However, if the participants had limited motor function, we also allowed them to play exergames while sitting in a stool or wheelchair. In addition, the single-player mode is the basis for using the 2-player mode. To let the participants fully understand and experience the exergame in a limited time, we only let them experience the basic mode, that is, the single-player mode.

The second part was to interview older adults with MCI regarding their experience and needs for exergames. It consisted of five broad, open-ended questions: (1) How did you feel after playing exergames? (2) Did you have any worries when playing exergames? (3) What do you hope to achieve by playing exergames? (4) What do you think the existing exergames need to improve? (5) How do you think exergames suitable for older adults should be designed?

### Data Analysis

Data analysis was guided by the methodology proposed by Giorgi [20], which represents a systematic approach to data collection, analysis, and synthesis suitable for scientific analysis. It emphasizes the importance of researcher engagement and reflection in these processes. The method developed by Giorgi [20] encompasses 4 steps. First, the transcripts were read in full to achieve immersion in the experiences and needs of the participants. Second, the transcripts were reread, and statements that conveyed the experiences and needs of the participants were recorded as units of meaning (codes). Third, codes related to the research objectives were grouped into themes. Finally, the units of meaning were investigated according to the research question, their basic structure was revealed, and these transformed units of meaning were synthesized into descriptive statements.

The data analysis was carried out in parallel with data collection. Two researchers (XC and DJ) listened to the audio recordings at the end of each interview to assess data saturation. Two researchers (XC and DJ) transcribed the audio recordings into text and a third researcher (HN) checked the two transcripts for consistency, and, if there were discrepancies, the recordings were replayed for verification and correction. To facilitate data management and analysis, transcripts were uploaded to the qualitative data management software NVivo 12. Two researchers (XC and DJ) simultaneously extracted, analyzed, and validated the information from the transcripts. In case of disputes, the aforementioned researchers discussed them, and qualitative research experts were consulted. The initial coding process for all collected data was independently carried out by the principal investigator (XC). The research team met weekly to discuss emerging themes; resolve differences in judgment; and review data analysis, coding, and results to facilitate interpretation of results as well as to ensure the credibility of the analytical process. The full dataset was reviewed by a member of this research team (HN) to validate the completed analyses. The research team concluded that the data reached saturation before recruitment ceased.

The trustworthiness of this study was evaluated according to four criteria: (1) credibility, (2) dependability, (3) confirmability, and (4) transferability. A variety of methods were used to ensure the trustworthiness of the study. These included taking reflective notes, triangulating data from multiple perspectives, conducting research team meetings, reporting on the study in detail, using purposive sampling, creating an audit trail, and utilizing verbatim quotes to support themes. In addition, the process of analyzing the data adhered to the standards required by the study’s design, and the final results were sent to all participants for validation and approval.

### Ethical Considerations

This study was approved by the ethics review committee of Nursing and Behavioral Medicine Research, School of Nursing, Central South University (E202397). All participants provided their written informed consent before enrollment in the study and retained the right to withdraw at any point in time. The data provided have been deidentified. No compensation was offered to the participants.

## Results

### Overview

A total of 21 interviews were conducted for this study. By the 18th interview, the data had become saturated, and we conducted 3 more interviews to ensure no new topics emerged. The average age of the study participants was 70.2 (SD 7.6) years, ranging from 60 to 85 years. Of the 21 participants, 17 (81%) were women participants. Regarding educational background, 24% (5/21) of participants had completed primary education, 33% (7/21) had completed junior high school education, 33% (7/21) had completed senior high school education, and 10% (2/21) had obtained a college education. All interviews were conducted in a face-to-face manner, with 76% (16/21) carried out in community settings and the remaining 24% (5/21) conducted in nursing homes. Complete demographic information is presented in Table 1.

**Table 1:** Participants’ characteristics (N=21).

Characteristics			Values
Age (y), mean (SD; range)			70.2 (7.6; 60-85)
Woman participants, n (%)			17 (81)
**Education level completed, n (%)**			
	Primary school	5 (24)	
	Junior high school	7 (33)	
	Senior high school	7 (33)	
	College level	2 (10)	
**Geographical location, n (%)**			
	Community	16 (76)	
	Nursing home	5 (24)	
MoCA^a^, mean (SD; range)			18.8 (3.6; 11-24)

^a^MoCA: Montreal Cognitive Assessment.

### The Experience of Older Adults With MCI Participating in Exergames

#### Attitudes Toward Exergames Vary

Most older adults with MCI (18/21, 86%) exhibited a positive attitude toward exergames, while a small number of them (3/21, 14%) were not interested in exergames.

Specifically, older adults with MCI found that exergames were easier to do than real exercise, less strenuous, easier to use, and provided an immersive experience that made them feel like they were doing real exercise:

Playing badminton with exergames is less strenuous than playing badminton in real life. We only need to use the remote control on our hands, which is not so tiring.

P10

This exergames is a good one to play at home. It is also a sport, better than outdoor sports, and it is convenient and good. It’s easy to use, easy to learn, and a little easier than the real thing. In reality, I may not catch the ball when playing badminton, but in exergames, as long as my hand moves, I usually hit the ball, which is very good for sports.

P13

In real life, I don’t know how to play badminton. But in exergames, I can play badminton, because I can control the ball well as long as I wave my hand at will.

P21

In general, it’s good, especially for me. Just when I was playing exergames, I wanted to go to the ground to pick up the ball, because I thought I was playing real badminton.

P4

A minority of older adults with MCI (3/21, 14%) were not interested in exergames due to several factors. First, they perceived exergames as primarily designed for younger individuals or children, thereby rendering them unsuitable for older adults. Second, they felt that the amount of exercise in exergames was excessive. Finally, some might have lacked interest in sports or have limited energy to engage in exergames:

Exergames are good, if you have time, you can still play this exergame. But I don’t want to play it anymore, because it’s for my son and grandson, not for us old people, and I don’t think it’s suitable for old people.

P12

It’s a little bit of exercise, which we certainly don’t normally do. We usually take a walk, we don’t run at this age, this sport is not suitable for us.

P8

I’m not very interested in these games, especially the ball game. I’m not really interested. I don’t have much energy for this.

P3

#### Both Entertaining and Interesting

Older adults with MCI found exergames enjoyable; they experienced a sense of accomplishment after successfully catching the ball and felt rejuvenated by interacting with the animated characters within the game. In addition, they felt that their mood would become happier and their spirit would become better after using exergames. In addition, they thought exergames were fun, so they would not be bored when they exercised:

I was happy when I caught the ball. I had a sense of accomplishment.

P12

We’re old people. It’s no fun playing with old people. After playing with the young people in this game, I feel that my mentality is much better, and I can feel younger in every way.

P16

After each activity, I will become happy and energetic, and I will not nod off. If I were sitting at home right now, I’d be dozing off. But now I feel refreshed after doing this exercise.

P17

I thought it was funny. I had a great time, ha-ha. Especially when I win.

P21

It’s fun. I feel comfortable when I play.

P1

This game is very interesting. I like it very much. It’s good for me, I love it. I think it’s interesting, it doesn’t get boring. Exercise can’t be boring, otherwise, you always feel that you are just there, no fun. Sports should be as fresh as this, or you can’t stick to it. In addition, there’s an opponent in the game to compete with me, which I think is fine.

P4

#### Promote Physical Activity and Exercise

In this interview, older adults with MCI suggested that exergames could encourage older adults to engage in physical activity and recognize its benefits over prolonged periods of sitting. In addition, they believed that using exergames could exercise various body systems, including training the brain, limbs, eyes, reaction speed, reaction ability, and coordination of various parts of the body:

If this is placed in the community activity center, it will certainly make older adults who have been sitting and playing cards increase their physical activity.

P15

Exergames are not only fun, but also can exercise the body. I usually sit at home on the sofa watching TV, not doing any physical activity, if there are exergames I can move my body.

P1

Exergames can exercise the body, especially the flexibility of the brain and hands, reaction speed and reaction ability.

P16

This game is good for our brains. While playing games, older adults can not only exercise their bodies, but also exercise the coordination and cooperation of the limbs and brain.

P20

This game is very good, it can exercise the body, especially for the strength of the arms. Old people just need to be active. In addition, exergames are good for the brain, eyesight and reaction capacity.

P2

Playing this exergame is beneficial for older people. Many older adults sit for a long time, which is not good for their health. After engaging in exercise, they can at least make their limbs more coordinated. In addition, we can also walk while playing badminton, which can play a role in exercising the whole body. Finally, by engaging in exergames, our muscles in the limbs are less likely to atrophy and arms become more flexible.

P6

#### Pass Time and Relieve Loneliness

Older adults with MCI found exergames as a way to pass the time and relieve loneliness. They thought that exergames can be used as entertainment tools to occupy their free time. Moreover, some older people would feel lonely because they lived alone, and playing exergames made their house lively:

If you live alone like me, when you are lonely or bored, you can do this exercise, which is very feasible.

P15

I like entertainment myself, not only to move the body, but also to spend time. I live alone, I can only watch TV when I rest, it is very quiet. I really want to go outside and have fun, not so lonely. The livelier, the better.

P16

Compared with real badminton, this is a pure entertainment, to pass the time. It suits us seniors who are retiring because we have nothing to do but pass the time.

P9

#### Conditions of Use Are Not Restricted

Older adults with MCI mentioned that the use of exergames was not restricted by factors such as location, number of participants, time, and weather conditions. First, exergames can be used both in the community and at home, but when used in the community, some older people may feel shy or embarrassed by being observed. Second, exergames can be used for both single training and double training, and they mentioned that they were willing to play exergames even if they were alone. Moreover, the use of exergames is not limited by time, older adults with MCI can use exergames at any time according to personal preference, and they can play alone without cooperating with others’ schedules. Finally, the use of exergames will not be limited by the weather conditions and eliminates the need to worry about not being able to participate in sports because of wind and rain:

Exergames can also be played at home. But it is not suitable for the community, because I’m not good at games, and there are a lot of people in the community, I feel uncomfortable and a little embarrassed by being observed.

P12

I can play badminton at home with my family through exergames, especially with my husband.

P14

I am willing to play this exergame by myself.

P1

This will allow us to exercise at home. Because the outdoor wind is not conducive to playing badminton, the stadium is far from home, and it is not convenient to go out when it rains. Exergames allow me to exercise at home and even do full-body exercises.

P8

This can be exercised indoors, especially at home and at any time. Even if there is no one to play with, I can play alone, and do not have to cooperate with other people’s time.

P7

I wouldn’t have to go out and play if I had an exergame. I just can’t walk because my knee is out of shape and I can’t move easily. And there are opponents in exergames, which can make me more interested in playing.

P2

### The Need for Exergames in Older Adults With MCI

#### Design Older Adult–Friendly Exergames

Older adult–friendly design should be centered around older adults, consider their needs for easy-to-understand products, and integrate them into the product conception and design [28,29]. Some older adults with MCI (6/21, 29%) thought that the pace and speed of the game were too fast and the operation was too complicated. A small number of older adults with MCI (3/21, 14%) preferred not to use game pads because they were complicated to use and easy to lose. Furthermore, some older adults with MCI expressed a desire for exergames to be designed to accommodate both seated and standing positions, to cater to the needs of older adults with physical limitations and to prevent prolonged sitting. Finally, older adults with MCI also hoped to design exergames that conform to Chinese culture, to facilitate their familiarity with and the use of exergames:

I’m old. This is a young man’s game. I can’t keep up. If the game is designed for older people, the speed and pace of the game should be slower. This is too complicated for me, we old people usually watch movies and TV for entertainment.

P12

This exergame is a bit complicated and difficult to use. I hope that as soon as I open it, I can enter the formal game matchmaking interface, and there will be no redundant selection interface, so that it will be faster for older people to use.

P13

I still don’t know how to use this gamepad. It would be much easier if we did not have to use a gamepad. In addition, it’s easy to forget where you put it, and you have to look for it everywhere before using exergames.

P20

It’s easier if I don’t have to use this gamepad and hold it all the time, because it’s complicated to use.

P21

Older people react more slowly, so the game should be designed to be slower and simpler so that we can react in time.

P9

This game can be played both sitting on a stool and standing up. But sitting all the time is not good for health, standing all the time is also not good, it is best to sit for a while and then stand for a while.

P1

Exergames should be designed to be able to sit on a stool to play the game, many older people’s legs and feet are not convenient, and it can also be convenient for disabled people with lower limbs to play this.

P5

I am not very good at using this gamepad. Not only are there many buttons on it, but all the words displayed on the gamepad are in English, which I can’t understand. It should be changed to Chinese. In addition, the whole exergame is very tedious to use, and there are many unnecessary steps, you should design a very simple and convenient game for older adults, so that we older people will take the initiative to play this game.

P4

#### Scientific Validity and Safety

Some older individuals with MCI expressed concern about the possibility of sustaining injuries while engaging in exergames. On the one hand, they were worried that they did not receive scientific guidance during the process of exercise and could cause injuries, such as muscle strains and sprained feet. On the other hand, many older people experienced various medical conditions (eg, high blood pressure, heart disease, knee problems, or vision impairment) and were worried about secondary injuries caused by these exercises. Consequently, all of these individuals hoped to ensure the scientific validity and safety of sports in the process of using exergames:

I’m afraid of getting hurt during sports now. Because I bought a grip strengthener last time, maybe the exercise was too intense, it hurt my cervical spine, and I still have pain in my cervical spine, which has never hurt before. I don’t know how to use the grip strengthener, I just grab it by myself. Although I thought I was doing well, using the grip 20 times a day, a few days later I injured my neck. We should not exert too much force in the process of exercise. Therefore, I now think about whether I will get injured when I exercise.

P4

For us old people, we are afraid that we are too old to do sports. Because some old people have high blood pressure and heart disease, they are afraid that he is too excited in the process of exercise, resulting in a sudden heart attack.

P7

We dare not play badminton now, because we are afraid that we will hurt our feet easily without protection. In addition, our age is not suitable for strenuous exercise, we can only gently do exercise at home.

P8

I used to do square dancing, but I haven’t danced for three to four years now because I have coronary heart disease and can’t be too tired. Besides, my knee joint is not good. It often hurts. This exergame is good, but I’m just afraid of hurting my joints or falling down. In addition, my heart can’t keep up, and I dare not do strenuous exercise, at most do some gentle exercise, such as Taijiquan and so on. Finally, I am afraid that we old people in the process of playing the game fell to the ground, resulting in a sudden death.

P10

I dare not play this exergame too much. Because I used to play video games when I was young, I even stayed up late playing games, which made my eyes not so visible. Thus, I don’t play video games now, for fear of hurting my eyes.

P18

#### Provide a Good Gaming Experience

Some older adults with MCI (3/21, 14%) were hopeful that future exergame developments would provide them with a good gaming experience. For example, older adults with MCI expected the actions of the game’s animated characters to align with their movements, expected to experience victories over defeats, and expected exergames to offer a diverse range of games for them to select and switch at any point:

Anyway, when my body moves, the game character inside the screen has to move, but if I don’t move, it shouldn’t move, its movement has to be consistent with mine. However, the movements of the animated characters in this game are not consistent with mine. I don’t like this routine of the game. It is not completely according to your actions, and there is no sense of reality.

P19

I’m playing ball with robots now. I always feel that I can’t win, so there is a sense of psychological loss.

P14

I hope you develop as many games as possible so that I can play different games. For example, I can play this game and then play that game, or play one game today and play another game tomorrow, and I will be happier.

P4

#### Exercise Physical and Cognitive Function

Older adults with MCI also expressed a desire for the future development of exergames to cater to their physical and cognitive needs. Regarding physical function, they aimed to exercise their bodies, improve the agility of their hands and eyes, prevent shoulder periarthritis and cervical spondylitis, improve the coordination of various parts of the body, help them keep healthy and fit, and strengthen their physique. Regarding cognitive function, they hoped to exercise their brains more during exergames, thereby enhancing memory function and reaction speed and mitigating the risk of Alzheimer disease:

I hope I can exercise my body through exergames, and at the same time I can use my brain more. If I don’t use my brain, my memory will get worse and worse.

P10

I hope to improve my reaction ability through exergames, as people’s reflexes in all aspects will slow down when they get old.

P11

I want to do this for fun, to pass the time and to exercise. Plus, hopefully this will help us lose weight and keep us in shape. Finally, more exercise can prevent Alzheimer’s disease.

P9

I hope to improve my hand-eye dexterity and reaction speed through this exergame. In addition, it would be better if I could exercise my legs through exergames.

P5

This exergame is good for body function. If I insist on playing it for a long time, it may help me prevent shoulder periarthritis and cervical spondylitis, and this activity is very good for the body.

P6

I hope to improve the coordination of all parts of my body through exergames, especially the coordination between the hands, eyes and brain. Besides, I can keep fit and strengthen my body through this.

P7

I mainly hope to exercise my knee through this. My knees hurt when I walk, but they feel better when I sit.

P3

#### Provide Support and Training

There was also a need to provide older adults with MCI with the necessary support and training in the use of exergames. Older adults with MCI may encounter various problems and difficulties in the process of playing exergames. At such moments, incentive mechanisms and game tips are needed to help them enhance their confidence and overcome difficulties. In addition, most older adults with MCI lacked experience and knowledge of exergames, so they wanted to get the necessary training before using exergames:

This game can add some encouragement so that it will be more interesting to play, and I will be more willing to play. For example, add some language communication, which will make people happier. The more you play, the more fun you have.

P16

It would be better to provide game tips, for example, telling me when to serve and receive the ball during badminton.

P1

I think voice interaction could be added to this exergame. For example, using voice to ask the user whether to play alone or in pairs, older adults can directly answer orally, which will be very convenient. In addition, the game should be very clear to tell me how to play the game, and what to play. Finally, it needs to provide as many game tips as possible, such as if I don’t know how to use the gamepad, the game automatically tells me which key to press at each step. Although I can understand it by reading the instructions, it will take a lot of time.

P4

To design suitable exergames for MCI seniors, it is necessary to ensure that the pace of the game is slow. At the same time, it is necessary to familiarize older adults with exergames and give relevant training to them before formal use.

P12

I think this exergame is great, but I would have been more receptive and enjoyed it if someone had shown me how to use it before the game started.

P6

## Discussion

### Principal Findings

To the best of our knowledge, this is the first qualitative study to have explored the experience and needs of older adults with MCI in China regarding exergames. This study included the perspectives of 21 older individuals with MCI and has contributed new knowledge to the exergaming domain through 2 themes: experiences of older adults with MCI regarding exergames and their needs for exergames. Given that exergaming is an innovative, fun, and relatively safe form of exercise [30], successful exergaming may be a promising approach to promote health in older adults with MCI. Therefore, this qualitative study is both timely and meaningful.

We begin to discuss our findings by reflecting on our methods. Our results were combined with our previous knowledge, experience, ideas, and opinions about our research topic, while we acknowledge that these subjective views could influence the results and conclusions of the research. Therefore, in order to maximize the credibility and confirmability of the findings, we thoroughly informed participants and obtained informed consent, established a non-judgmental and open environment during interviews, adopted active listening and open question techniques to encourage honest and comprehensive answers, and engaged in continuous reflection during data collection and analysis. We also conducted regular research team discussions in the process of data analysis. All differences and issues were thoroughly discussed and resolved through collaborative efforts within the research team, with all authors confirming the findings in this study. Therefore, we identified our themes in the process of reflection. It is worth noting that the principal investigator (XC) is a doctoral student with a background in geriatric nursing and digital health research, who received training in systematic qualitative research methods and completed a six-month internship in the Sports Rehabilitation and Medical Robotics team at the Ningbo Institute of Materials Technology and Engineering. As a result, her knowledge and insights in these areas increase the richness of the data, improve the reliability of data collection, and deepen the interpretation of the findings. At the same time, the authors (HN and HF) are experts in geriatric nursing, digital health, qualitative research, and nursing informatics research. The work and research experience of the other coauthors in this field also enhanced the analytical process by introducing new perspectives.

In addition, this study used the COREQ checklist to improve reliability. To enhance confirmability, researchers actively engaged in thorough self-reflection, maintaining a high level of honesty and openness. To support transferability, we provided a detailed description of the study background, participant inclusion criteria, participant demographics, data collection and analysis procedures, and findings to enable readers to assess the applicability of our findings to other settings. Reflective note-taking, interview scheduling, and minutes of supervisory research group meetings provided audit clues for the analysis. The paper also provided many quotes from different participants and used verbatim quotes to support the themes to provide authenticity.

### The Experience of Older Adults With MCI Participating in Exergames

Most older adults with MCI expressed a positive attitude toward exergames, citing them as a fun, enjoyable, and novel experience. Previous qualitative studies have also demonstrated pleasurable experiences when participants engage in exergames [31,32]. These previous findings, along with our own, suggest that exergames may be a promising tool for creating fun during physical exercise. However, it should be noted that not everyone is interested in exergames, and not all exergames are suitable for older people. Our team previously published a systematic review and qualitative meta-analysis of older adults’ experiences with exercise gaming programs [17]. The reasons we identified for older people’s lack of interest in exergames are as follows. First, lack of ability to use exergames, due to age and health-related factors (vision, hearing, motor skills, or cognitive impairment). Second, lack of experience in playing exergames, leading them to worry that they cannot understand and play such games correctly. Third, older people in East Asian countries (such as China, Japan, and South Korea) may feel embarrassed when using exergames due to potential observation or judgment by others. Finally, most existing exergames require a high degree of flexibility and adaptability, which may not be entirely suitable for older people. Nonetheless, most barriers can be addressed by designing exergames for different target groups and giving older people enough time to train and familiarize themselves with exergames.

Our findings found that the vast majority of older adults with MCI believe that exergames are both entertaining and interesting. The possible reason is that exergames are a combination of sports and games, which can not only allow older adults to enjoy immersive game experiences but also encourage them to participate in sports through games, thereby increase the entertainment and fun of their sports [33-35]. Many previous studies have also concluded that exergames are an entertaining, interesting, and attractive form of physical exercise [36-38]. In addition, a recent systematic review and meta-synthesis of 11 studies [39] found that older adults view exergames as a pleasurable experience and suggested that health care professionals and aged care centers may consider using exergames to encourage older adults to participate in and adhere to regular exercise [39].

Physical activity is an effective, cost-effective, and nonpharmacological intervention to reduce the risk of dementia [40,41]. Relevant studies have shown that physical activity can substantially improve the cognitive function, physical function, and quality of life of patients with MCI [42,43]. However, older adults with MCI have low levels of physical activity and are sedentary [44,45]. A cross-sectional survey of global aging and adult health conducted by the World Health Organization in 6 low- and middle-income countries, including China, showed that people with MCI were 1.28 times more likely than people without MCI to not meet internationally recommended physical activity standards [46].

Our findings indicated that older adults with MCI believe that exergames can encourage them to engage in physical activity and recognize its benefits compared to prolonged sitting. This result may help address low levels of physical activity and sedentary behavior among older adults with MCI. Moreover, our results are consistent with previous studies, which also suggest that exergames can encourage and motivate older adults to participate in physical activity [8,15,47].

In addition, exergames are expected to be an important way to improve the mental health of older adults. Our results suggest that some older adults with MCI believe that exergames can alleviate their loneliness. The results are consistent with previous studies. An intervention study involving 20 healthy older adults indicated that exergames may help address social isolation and loneliness among this population [48]. In addition to loneliness, other studies have suggested that exergames can improve other aspects of mental health, including apathy, anxiety, and depression [49,50]. A systematic review of 10 randomized controlled trials showed that exergames had a positive effect on mental health in older adults, reducing the incidence of apathy, anxiety, and depression among older adults [50]. A systematic review of 18 randomized controlled trials with a sample size of 1023 individuals aged >60 years revealed that the longer duration of the exergame intervention, the greater improvement in depression among older adults [49]. Furthermore, a randomized controlled experiment [26] involving 55 older patients with cognitive impairment showed that exergames could substantially decrease depression scores and improve depressive symptoms in these individuals. Finally, our findings indicated that the utilization of exergames is not restricted by factors such as location, number of participants, time, and weather conditions. This is consistent with previous findings. A systematic review and meta-analysis of 18 randomized controlled trials [49] has proposed that the use of exergames are not limited by sports facilities, time, place, space, meteorological environment, or the number of people. Multiple studies have also demonstrated that exergames can be used in different settings, such as home environments, hospitals, communities, and nursing homes [15,49,51]. Moreover, one study suggested that exergames can not only provide individual training but also can be performed in small groups [14].

It is worth noting that we encouraged the participants to express themselves freely and fully respect the opinions they express. However, in addition to aspects related to the use of exergames for older adults with MCI, our study found that participants were less likely to report other multidimensional health aspects, such as social interactions and quality of life. Considering the overall health of older adults, especially in the context of MCI, it is crucial to explore how exergames can contribute to these other dimensions of health beyond cognitive and physical exercise alone. Therefore, it is suggested that future studies should increase the investigation on this aspect, which can provide more comprehensive guidance for the design of exergames. In addition, it is recommended to pay more attention to the integration of exergames with other multidimensional health aspects in the design and development of exergames, as well as the implementation of exergame intervention and promotion, which will help improve the overall health of older people with MCI.

### The Need for Exergames in Older Adults With MCI

First, age-friendly exergames should be designed, which is particularly important to improve older people’s exergame experience and participation enthusiasm [15,52]. The results of our research found that some individuals with MCI felt that the pace and speed of the game were too fast, and that the controls in the game and the use of the gamepad were too complex. The possible reason for this is that the target users of existing exergames are teenagers and young people, who have too high requirements for game skills, and the game design is not only too complicated and difficult but also lacks attraction, entertainment, and interaction [53,54]. In addition, the experience of older adults with MCI with exergames also influenced their experience. One study suggested that older adults lacked experience in using exergames, which makes it difficult for them to get familiar with them, results in their insufficient acceptance and lack of motivation to use exergames [55]. As a result, existing exergames (such as the Wii and Kinect) may not have been designed to be used by older people [17]. Therefore, it is imperative to design and develop age-appropriate exergames that cater to the specific needs and characteristics of older adults with MCI to enhance their acceptance and engagement in these activities.

Second, it is essential to guarantee the secure and scientific use of exergames by older adults with MCI. Most of the existing exergames are designed for entertainment, without considering the principles of sports [15], and lack scientific training standards and sports programs, which limits older adults from obtaining the maximum health benefits from exergames [14]. According to a previous study [56], one of the most important reasons for the poor rehabilitation effect of exergames on patients with musculoskeletal back and neck pain is that existing exergames are designed for entertainment. Therefore, future studies should target older adults with MCI, take health benefits as the purpose, and add rigorous exercise programs with scientific validity to exergames to better meet the needs of older adults with MCI. In addition, exergames may cause some harm to older adults with MCI. For example, older adults with MCI may be prone to visual and hearing impairment due to prolonged exposure to high-brightness screens and noisy game sounds [57]. There are also many older individuals with various diseases, particularly high blood pressure and heart disease, and at times, even multiple diseases coexist. This may result in potential accidents in the process of playing exergames. Therefore, it is necessary to ensure the safety of older adults with MCI when engaging in exergames to prevent any potential injuries.

Third, it is necessary to ensure that older people with MCI have a good gaming experience during the use of exergames. Older adults with MCI may have difficulty understanding and using exergames due to learning and cognitive decline. The complex project function and level design in exergames increases the learning and cognitive challenges of older adults with MCI, often causing them to have doubts when using exergames, which can easily make them feel frustrated and withdraw, thus reducing their enthusiasm to participate in exergames [28]. However, the mode can be simplified to make older adults directly understand the operation logic and rules of exergames and reduce the possibility of their wrong operation, to improve the usability of exergames and optimize the experience of exergames for older individuals [28]. Exergames also do not always accurately track and record the movement of older adults, which makes gameplay frustrating [48]. Exergames that incorporate speed elements may further exacerbate the frustration of physically restricted users, reducing their self-efficacy, motivation, and enjoyment of exergames [48]. Therefore, this aspect should be taken into account when designing exergames for older adults with MCI.

Fourth, exergames need to exercise the physical and cognitive functions of older adults with MCI. MCI is characterized by a decline in cognitive function that exceeds the expected level for an individual’s age and education level, but with no substantial disruption to activities of daily living [58]. In addition, increasing age is associated with declines in cognitive and physical function [59]. Previous studies have also shown that physical functional parameters, such as gait speed and grip strength, correlate with cognitive function in older adults [60-62]. Cognitive function and physical function are major determinants of quality of life in older adults, and any reduction in these 2 factors may affect the ability of older adults to perform activities of daily living. Previous systematic reviews have indicated that exergames are one of the most effective exercise interventions to improve health outcomes (such as cognition and function) in older adults [9,10]. Therefore, to avert the decline in cognitive and physical functions in older adults with MCI, the design of exergames targeted at this population should focus on the exercise of physical and cognitive functions.

Finally, older adults with MCI need to be provided with the necessary support and training in the use of exergames. Older adults with MCI may easily feel frustrated and disappointed when they encounter various problems or difficulties during the gaming process. Therefore, timely support and guidance should be provided to enhance the self-confidence of older adults, overcome the “digital divide,” and ensure their continued participation in exergames [28]. Two studies [32,63] have proposed that frequent and positive feedback from people around them can increase the enthusiasm of older adults who are just beginning to participate in exercise, effectively improve their confidence, create a sense of pride and achievement, and improve their acceptance of exergames and participation motivation. Furthermore, most older individuals lack the experience and knowledge concerning exergames [64], and necessary training should be provided for older adults with MCI before using exergames, including theoretical instruction, a hands-on demonstration of operation, and practical experience for the participants.

### Limitations

There are some limitations in this study. First, this study only focused on the experiences and needs of older adults for exergames, and future research should also explore how other stakeholders view exergames, such as family members, caregivers, and health care professionals. Second, our sample size was relatively small, but our interview data reached saturation, indicating that adding new participants will not provide any additional or valuable insights. At the same time, Patton [65] also emphasized that sample size is not as important as other methods in qualitative research. Third, most older adults lack access to new technologies, and only an initial gaming experience was provided for older adults with MCI in this study, which may have limited their comprehension of exergames. However, initial saturation results have been achieved. Fourth, the uneven gender distribution was also a limitation of our study. Out of the total participants, 17 were woman participants while only 4 were man participants, with a substantial overrepresentation of woman participants. This may be because woman individuals are more prone to Alzheimer disease [66]. Fifth, the location of participants was not uniform. This is because the severity of cognitive impairment in older adults in nursing institutions is generally high, while our study only needs older adults with MCI, making it challenging to recruit suitable participants in nursing institutions. Sixth, the selection of exergames in this study is limited to Nintendo Switch Sports on Nintendo Switch, and there should be a wider range of games and platforms to choose from, so that the participants can better understand the nature of games. Finally, this study was limited to a community and nursing home in Changsha, Hunan Province, China, so the findings may have limited generalizability to other settings and countries.

### Conclusions

This study offers an interpretative understanding of the experience and needs of older adults with MCI in relation to exergames. The experience of older adults with MCI with exergames included 5 parts: attitudes toward exergames vary, both entertaining and interesting, promote physical activity and exercise, pass time and relieve loneliness, and conditions of use are not restricted. The needs of older adults with MCI for exergames include the desire to design older adult–friendly exergames, ensure science and safety in the process of sports, provide a good gaming experience, exercise physical and cognitive functions, and provide support and training. This study can provide qualitative evidence to guide the development of exergames tailored for older adults with MCI. Future studies are needed to extend the effectiveness of our findings to a larger population of older adults.

## Abbreviations

COREQ: Consolidated Criteria for Reporting Qualitative Research
MCI: mild cognitive impairment

## Data Availability

The datasets generated or analyzed in the course of this study may be provided by the corresponding author on reasonable request.
